# Protein design, generative AI and biological security

**DOI:** 10.3389/fmicb.2026.1817535

**Published:** 2026-04-01

**Authors:** Maximilian Brackmann, Sophie Reiners, Masja Hoogendoorn, Michel Moser

**Affiliations:** 1 Spiez Laboratory, Spiez, Switzerland; 2 Center for Security Studies, ETH Zürich, Zürich, Switzerland; 3 ETH Zürich, Zürich, Switzerland

**Keywords:** biochemistry, biological security, computational biology, dual-use, generative AI, medical countermeasures, protein design

## Abstract

Artificial intelligence-driven protein design has fundamentally changed what is possible in protein engineering. Deep learning models can now generate entirely novel sequences that fold into defined structures, enabling advances in therapeutics, vaccine development, and industrial biotechnology. For biosecurity specifically, designed proteins offer new opportunities: capturing and detecting biological agents, developing novel binders against viral surface proteins, and accelerating pandemic preparedness. Yet the same capabilities introduce new risks. AI-generated proteins may be functionally equivalent to known toxins while sharing little sequence similarity, rendering current homology-based screening blind to such designs. The wide availability of open-source tools further lowers the barrier to misuse. Mitigation requires layered strategies that together can deter misuse without stifling innovation. Here, we review the current landscape of generative protein design, assess its dual-use implications, and discuss proportionate mitigation strategies that balance open scientific progress with biosecurity.

## Introduction

Artificial intelligence has rapidly transformed protein science, culminating in the 2024 Nobel Prize in Chemistry for work on AI-based protein structure prediction and design (The Nobel Prizes, 2025). This recognition marked a turning point: protein sequences can now be translated into accurate three-dimensional structures, and, increasingly, entirely new sequences can be designed *in silico* with predefined structural or functional properties (Koh et al., 2025). These developments are reshaping protein engineering while simultaneously raising important questions for biosecurity.

Historically, protein engineering focused on modifying naturally occurring proteins. Experimental approaches such as directed evolution enabled incremental optimization of stability, activity, or specificity through iterative mutation and selection (Sellés Vidal et al., 2023). Early computational methods for protein structure prediction mirrored this paradigm: they relied on experimentally solved protein structures and homology modeling or physics-based energy functions to predict the impact of mutations (Waterhouse et al., 2018). Consequently, their applicability was largely restricted to proteins with close structural homologs.

## Emergence and impact of deep learning in protein design

The number of experimentally solved structures and advances in deep learning alleviated this requirement. The Critical Assessment of Techniques for Protein Structure Prediction (CASP) challenge demonstrated that, from 2018 onward, deep learning–based approaches consistently outperformed traditional methods (Kryshtafovych et al., 2019). Rather than relying on single templates, these models infer statistical relationships between amino acid sequence and three-dimensional structure from large datasets of known proteins, enabling accurate structure prediction even in the absence of close homologs (Winnifrith et al., 2024).

The ability of deep learning models to learn joint patterns linking sequence, structure, and, to a limited extent, function has profoundly expanded the scope of protein engineering. It has enabled not only reliable structure prediction but also the rational design of new proteins with increasing confidence in their predicted properties.

## Core deep learning tasks: structure prediction and inverse folding

Deep learning models in protein science can be broadly divided into discriminative and generative approaches. Discriminative models learn conditional probability distributions and focus on predicting the most likely output for a given input. AlphaFold 2 is the canonical example: it predicts a protein’s three-dimensional structure from its amino acid sequence but does not generate new sequences (Abramson et al., 2024).

Generative models, by contrast, aim to learn the joint distribution of sequences and structures. This capability enables *de novo* protein design. In so-called inverse-folding or “fold design” tasks, models generate amino acid sequences expected to fold into a predefined backbone structure. Early inverse-folding approaches relied on iterative cycles between sequence generation and structure prediction to refine designs, while more recent models predict sequence and structure jointly or employ independent structure prediction models for confirmation (Albanese et al., 2025; Listov et al., 2024). Two principal strategies are used in protein design: *Ab initio* approaches create entirely new sequences not observed in nature that fold into novel structures, whereas template-based approaches modify existing proteins to alter stability, activity, or specificity (Koh et al., 2025). Both strategies have benefited substantially from deep learning–based generative models.

## Generative modeling frameworks and current limitations

Protein design has progressed through several generations of generative modeling frameworks (Tang et al., 2024). Early variational autoencoders and generative adversarial networks demonstrated that protein sequences could be learned and sampled but often lacked the precision required for complex design objectives (Tang et al., 2024). More recently, autoregressive transformer models and diffusion-based approaches have achieved substantially higher design accuracy (Chu et al., 2024; Jendrusch and Korbel, 2025; Stark et al., 2025). Diffusion models such as RFdiffusion can generate realistic protein backbones and scaffolds for functional motifs, enabling the design of binders and other functional proteins with experimentally validated success (Das, 2025; Stark et al., 2025; Tang et al., 2024). Despite rapid progress, important challenges remain. Accurately modeling flexible or dynamic regions, accounting for cellular environments, and translating *in silico* designs into robust experimental outcomes continue to limit success rates (Lisanza et al., 2025). These challenges are particularly pronounced when moving from fold design toward reliable function design (Chu et al., 2024).

## From fold design to function design

Function design extends beyond specifying a target structure to generating proteins that carry out a desired biochemical activity. This can involve embedding known functional motifs into designed scaffolds or attempting to create entirely new functions from first principles. While significant advances have been made, especially for binding and simple catalytic activities, reliable *de novo* function design remains a major challenge (Chu et al., 2024).

## From computation to experiment

Advances in algorithms and computing power have made *in silico* protein design rapid and inexpensive. Experimental validation, however, remains labor-intensive (Albanese et al., 2025). Designed proteins must be chemically synthesized or expressed in biological systems. While peptide synthesis is increasingly efficient, recombinant expression in bacterial or eukaryotic systems remains the method of choice for proteins longer than approximately 100 amino acids. High-throughput screening often relies on cell-free expression, whereas larger-scale production requires microbial or cell-culture–based systems, which are costly in terms of time and labor. In most cases, either the protein itself or the nucleic acid sequence encoding it must be ordered, linking computational design directly to existing synthesis and screening infrastructures.

## For what do we need new or better peptides and proteins?

Peptide and protein therapeutics are among the fastest growing classes of therapeutics, in part due to their versatility and potential high affinity to target proteins (Kinch et al., 2022; Stark et al., 2025; Wang Y. et al., 2025). The high specificity of antibodies can be exploited to target certain cell types e.g., in cancer therapy (Carter and Rajpal, 2022). Some slightly modified peptide therapeutics for the treatment of obesity constitute the best-selling drugs in companies’ portfolios (Carter and Rajpal, 2022; Kristensen et al., 2019; Precedence Research, 2025). Currently, most peptide and protein therapeutics are administered intravenously as they usually are degraded by the digestive system, however, orally available peptides are being explored (Anderson et al., 2020; Heinis, 2011; Rhodes and Pei, 2017). These examples highlight the versatility and potential of protein and peptide therapeutics. They also highlight that there is room for improvement to increase stability and bioavailability.

## What can be done with protein design algorithms?

Proteins generated with the assistance of generative models are expected to greatly advance our understanding of biology. They are enabling advances across different fields in medicine, technology and sustainability. The creation of optimized protein scaffolds, such as truncated or hyper-stable versions of enzymes, would facilitate research and drug development. Designing better binders to modulate cellular activity or precisely inhibit pathogens present other potential use cases. Similarly, design of optimized antibodies for treatment of different diseases is a field of great potential. SKYCovione, the first computationally designed protein-based vaccine, was approved in South Korea and the UK, and listed by the WHO for emergency use in 2023 (Em, 2023). It highlighted the important role of protein design in pandemic preparedness. To increase sustainability in the chemical industry, generative models could help create proteins with improved catalytic properties that promote greener chemical synthesis or the design of plastic-degrading enzymes.

## What are the opportunities for biodefence and biosecurity?

Designed proteins can support the enrichment of biological agents for their subsequent detection by capturing the agent. In this way, protein design tools can support biosecurity by advancing detection methodology. Similarly, designing proteins that bind to viral surface proteins could provide new treatment options, with particular value for pandemic preparedness. Some groups have already developed specific binders against influenza virus or SARS-CoV-2 (Cao et al., 2020; Watson et al., 2023).

## Which risks do these models pose?

Generative algorithms for protein design have already mastered the art of mimicking nature’s existing repertoire. However, the same models that simulate 500 million years of evolution can be redirected to bypass it (Hayes et al., 2025). They can be used to create entirely novel toxins, engineering variants that evade current diagnostics, or drastically amplifying the potency of known biological agents. Designed stable protein variants might persist longer in the environment or withstand the low pH in the stomach when ingested.

One vulnerability lies in our current biosecurity screening infrastructure. Present methods for controlling the export and synthesis of nucleic acids or proteins rely almost exclusively on homology (sequence similarity) to known agents. Because AI-generated proteins may only remotely resemble natural sequences, current screening software is often blind to these “*de novo*” designs (Wittmann et al., 2025). Even if a designed protein is structurally and functionally identical to a known and controlled toxin, it can remain undetected at the sequence level, allowing restricted agents to be ordered and synthesized without triggering a license requirement or security alert.

Additionally, many detection methods for proteins, e.g., ELISA or mass spectrometry rely on structure or sequence similarity or even identity for confident identification of a protein. In their current form, they will unlikely detect newly designed proteins. However, functional assays would still detect them as they are not based on sequence similarity but rather mimic host responses and measure the biological activity. They help assess the potential toxicity of a protein, provided the novel protein uses a mode of action similar to a known toxin. If the toxicity of a protein toxin is enhanced, it may be correctly identified, but the standard treatment regimen might not be sufficient to counteract the activity of the toxin.

Most protein design tools are openly available, providing access to a wide population including individuals with potential malicious intent. Advances in tool design and integration with other software will provide easier access to these tools and at the same time lower the threshold for using them.

## What can we do to mitigate the risks?

Protein design software itself doesn’t pose a risk. The risk only manifests once the proteins are produced and made available. Nevertheless, these tools can still contribute to risk mitigation strategies.

Safeguarding the use of generative algorithms in protein design can occur at three levels. (a) The sequence of the designed protein is screened, (b) its nucleic acid sequence is screened before it is being produced, or (c) the design software itself implements security measures (Table 1).

**TABLE 1:** Possible security measures that can be implemented as safeguards.

*In silico* protein design	DNA synthesis level
Watermarking digital sequences (Chen et al., 2025; Wang M. et al., 2025; Zhang et al., 2025b)	Watermarking synthetic DNA (Berezin et al., 2024; Zhang et al., 2025a)
Models: access control and usage logging (The Nuclear Threat Initiative, 2024; Wheeler, 2025)	Synthesis providers: access control and usage logging
Model screening and refusal mechanism (The Nuclear Threat Initiative, 2024)	Sequence screening (Hunter, 2024; Wittmann et al., 2025)
Targeted unlearning and restricted training data (King et al., 2025; The Nuclear Threat Initiative, 2024; Wang M. et al., 2025)	Encrypted sequence logging (Baker and Church, 2024)
Safety model alignment, anti-jailbreak and output filtering (Wang M. et al., 2025)	Regulation of DNA synthesizers (The Nuclear Threat Initiative, 2023; Wheeler, 2025)
Audit trails of design metadata (The Nuclear Threat Initiative, 2024; Wheeler, 2025)	

Many DNA sequence providers check the sequences of their orders against a database of “sequences of concern.” If the ordered sequence is compared to a DNA sequence database and the best-hit is a sequence of concern, it would be flagged for inquiry. The detection of sequences which are unrelated but functionally identical to listed proteins, e.g., by export control regimes, poses a challenge as the listed proteins are not identified in sequence homology searches. However, these designed proteins tend to have sequences, which are not found in nature, which in itself can be considered a flag to have a closer look at the ordered sequence.

Additionally, the integration of structure prediction algorithms in the screening process – potentially only for flagged orders having no sequence homology to any non-problematic protein – and a subsequent homology search based on structural homology can provide additional clues on the nature of the order. Strengthening and optimizing screening software is one approach to better detect proteins of concern. In a recent study, screening system software for DNA sequences was improved to better detect proteins of concern even after their sequences have been modified using generative protein design tools (Wittmann et al., 2025). Screening systems for protein orders could be developed along the same lines.

These measures would work on proteins that have been designed already and are to be ordered. Another approach is the built-in of safeguards into AI models themselves (Zhang et al., 2025b). One possible mitigation measure is the targeted unlearning of harmful knowledge from a pre-trained model or the exclusion of sensitive training data in the first place (King et al., 2025; Wang M. et al., 2025). Therefore, the software would be worse at predicting malicious proteins than at predicting other proteins. The models could also include security measures to e.g., not work with toxins by incorporating sequences of concern, to compare queries against. It could be set up that it recognizes potentially harmful requests so that the requested design is blocked (Wang M. et al., 2025).

## What if someone produces and uses harmful material?

To reliably detect maliciously designed proteins, laboratory detection methods will have to change. The look-out for known toxin proteins in suspicious samples remains vital, but at the same time, untargeted and unbiased methods, specifically investigating previously unknown proteins in samples is becoming essential. Functional assays that e.g., provide evidence for the enzymatic function of a toxin are more robust to challenges posed by designed proteins, and consequently will become more important (Kalb et al., 2006; Rosen et al., 2017; Wang et al., 2016). Additionally, as seen in many other fields, advances in algorithms and more powerful hardware for unbiased sample characterization will enhance detection capabilities. However, careful evaluation and interpretation of the resulting data remains crucial for informed assessments on sample composition (Yilmaz et al., 2024).

Finally, once harmful material is detected and characterized, investigations on the origin of the material may start. Watermarking is an example of a built-in safeguard, where the generated outputs can be traced and audited. These watermarking strategies can embed identity tracing codes in protein design models which would make it possible to trace AI-designed proteins back to their source (Zhang et al., 2025b). While elegant, this approach has limitations. If the protein is altered, the watermark might be changed and harder to trace back. Furthermore, many software tools currently available are open-source and do not require user authentication. Logging user queries could deter misuse and allow authorities to retrospectively identify the designer of a protein used in an attack, but it also raises privacy considerations. Notably, nucleic acid synthesis providers already store records of produced sequences. This information is critical for forensic investigations involving designed proteins (Baker and Church, 2024).

## Discussion

The rapid advancement of protein design tools holds great potential for innovation but simultaneously introduces biosecurity challenges. As AI models get increasingly better and their predictions more accurate, security measures must evolve accordingly. Traditional approaches might no longer be sufficient and more targeted strategies are needed. To effectively detect and flag AI-generated proteins, existing screening tools should be optimized or replaced by new, more targeted software tools.

Currently, most screening processes rely on sequence homology-based screening which makes it difficult to detect AI-created proteins. A better approach would be the development of structure-based homology prediction tools. However, these new screening technologies are costly and computationally intensive to run, potentially slowing down the process of screening (Hunter, 2024). Moreover, integrating built-in safeguards into AI models will likely increase development and operational costs (Wang M. et al., 2025).

When considered in isolation, individual risk mitigation strategies might not seem very effective. Yet, in combination they can have a deterring effect. For example, integrating embedded safety and security checks in the design software and logged access to it, enhanced screening processes for AI-generated sequences or logging the generated sequences could collectively strengthen biosecurity measures.

More sophisticated protein design tools and their wide accessibility through open-source platforms, will make it easier for a broader range of users to access these models. Producing a toxin from an AI-generated sequence still requires expertise, resources and the right equipment and infrastructure. To locally run these models a powerful computer is necessary. To produce and purify a protein, a laboratory with basic equipment is needed. However if this process is combined with an increase in automation of protein production, as seen in biofoundries, it could open up a new level of risk.

The scientific community is increasingly aware of the risks such models could bring along. Initiatives like the 2024 “Responsible AI x Biodesign” reflect a growing commitment for the responsible development of AI technologies in protein design (Responsible Ai x Biodesign, 2025). Researchers in the field have warned that “Protein-design technology is vulnerable to misuse for producing dangerous biological agents,” raising awareness about biosecurity risks in protein design (Baker and Church, 2024). To stay ahead of new risks, regular monitoring and risk assessment of these models is necessary. Initial efforts are underway to develop a framework for the systematic assessment of AI-tools and their misuse potential (Rand, 2025).

While biosecurity risks must be addressed with urgency, it is required to do so without hindering the transformative potential of these models. Ultimately, we must strike a precise balance between fostering open innovation and implementing robust security safeguards.
